# Supplementary material for: Hyperbolic Neural Population Geometry Benefits Computation
Source: ArXiv. 2026 Jun 8:arXiv:2606.10238v1. Preprint. [Version 1] (PMC13278257)
Supplement: Supplement 1 [file NIHPP2606.10238v1-supplement-1.pdf]

Supplementary Materials

A. Limitations 14
B. Table of Notations 15
C. Related Works 16
D. Additional Background 17
D.1. Visualization of δ-thin Triangles 17
D.2. Properties of Hyperbolic Geometry 17
E. Proof of Main Text Results 22
E.1. Proof of Proposition 2.2 22
E.2. Proof of Theorem 4.2 23
E.3. Proof of Theorem 4.8 27
F. Simulation Details 33
F.1. Details of Pattern Completion 33
F.2. Multiple Instance Learning 33
F.3. Machine Learning Layers 34
G. Additional Simulations 36
G.1. Additional Simulation on Pattern Completion 36
G.2. Additional Simulation on Empirical Hyperbolicity 36

LLM Usage Disclosure. We use AI tools such as LLMs to aid with polishing writing including improving clarity and grammar. All the technical results are original contributions by the authors.

A. Limitations

Our tuning-curve model assumes single-field neurons and uniform peak firing rates. However, these properties can be violated in large environments (Rich et al., 2014). In the neuroscience setting, it would also be interesting to go beyond the hyperboloid model to calculate memory capacities when the embedding dimension far exceeds the dimensionality of the hyperbolic manifold. It is unclear whether the hyperbolic structure we identify is a consequence of more general constraints in biology or a phenomenon specific to hippocampal place cells. Distinguishing these would clarify whether our framework generalizes to other brain regions. For instance, hyperbolic geometry has also been reported in the olfactory and visual systems (Zhou et al., 2018; Lee et al., 2024).

B. Table of Notations

To distinguish between hyperbolic and Euclidean points, we use regular font for Euclidean variables and bold font for hyperbolic variables.

Table 2. Mathematical Notations and Symbols

Symbol	Description
Indices and dimensions	
N	Number of neurons (index $i \in [N]$; Section 2)
M	Number of stored patterns or grid points (index $\mu \in [M]$)
d	Latent hyperbolic embedding dimension ($\mathbb{H}_\kappa^d$)
D	Dimension of stimulus domain $\mathcal{S} \subset \mathbb{R}^D$
Stimulus space, spikes, and tuning	
$\mathcal{S}$	Stimulus space (e.g. $\mathcal{S} = [0, L]^D$ in Theorem 4.2)
s	Stimulus (location)
$n = (n_1, \ldots, n_N)$	Spike counts in a window (Section 2)
$\lambda_i(s)$	Tuning curve of neuron i (Equation (2.1))
$\lambda(s)$	Population rate vector $(\lambda_1(s), \ldots, \lambda_N(s))$
$s_{ik},\ \sigma_{ik},\ \lambda_{ik}$	Center, width, and scale of the k-th field of neuron i (Equation (2.1))
K	Number of place fields per neuron (often $K = 1$; Equation (2.1))
$\lambda_{\max}$	Peak firing rate (uniform-field simplification; Equation (2.1))
ρ	Intensity of Poisson field centers (Theorem 4.2)
Riemannian hyperboloid model	
$(\mathcal{M}, g)$	Riemannian manifold with metric g (Hadamard in our setting)
$T_x\mathcal{M}$	Tangent space at $x \in \mathcal{M}$
$d_g(x, y)$	Geodesic distance between $x, y \in \mathcal{M}$
$\mathrm{Exp}_x(\cdot)\ ,\ \mathrm{Exp}_x^{-1}(\cdot)$	Exponential map and inverse at x
$\mathbb{H}_\kappa^d$	Hyperboloid (Lorentz) model, curvature $\kappa < 0$
$\langle \cdot, \cdot \rangle_L$	Lorentz (Minkowski) inner product
$\boldsymbol{o}$	Origin (reference point) on $\mathbb{H}_\kappa^d$
$\boldsymbol{v}$	Query / state on $\mathbb{H}_\kappa^d$
v	Log-coordinates at $\boldsymbol{o}$: $\mathrm{v} = \mathrm{Exp}_{\boldsymbol{o}}^{-1}(\boldsymbol{v})$
$\boldsymbol{\sigma}$	Noise variance applied on $\boldsymbol{v}$
Memory patterns and maps	
$\psi^H : \mathbb{R}^N \to \mathbb{H}_\kappa^d$	Feature map from firing rate to hyperbolic space (Section 4)
$\psi^E : \mathbb{R}^N \to R^d$	Feature map from firing rate to Euclidean space (Section 4)
ξ_μ	μ-th stored memory pattern in $\mathbb{R}^d$
$\boldsymbol{\xi}_\mu$	μ-th stored memory pattern in $\mathbb{H}_\kappa^d$
$p(\mu \mid n)$	Posterior over memory index given spikes (Section 4)
$w_\mu(\boldsymbol{v})$	Softmax weights in the Karcher-flow update (KFM)
$H(\boldsymbol{v})$	Karcher-flow update rule (KFM)
Hyperbolicity	
d_{ab}	Semi-metric on stimuli from population inner products (Section 4)
$\Delta(s_a, s_b, s_c, s_d)$	Empirical Four-point hyperbolicity (Theorem 4.2)
δ	Hyperbolicity scale (Theorem 3.2)
L	Stimulus space diameter $\mathcal{S} = [0, L]^D$ (Theorem 4.2)

C. Related Works

Associative Memory Models. The foundations of associative memory models were established in the works of (Amari, 1972; Nakano, 2007; Amari & Maginu, 1988; Hopfield, 1982). The classic Hopfield network (Hopfield, 1982) is one of the most well-studied associative memory models due to its energy-based structure and theoretical accessibility. Recent studies (Krotov & Hopfield, 2021; Ramsauer et al., 2020) substantially increased the storage capacity of Hopfield networks and generalized associative memory models to continuous domains. More recently, modern Hopfield networks have been shown to be closely related to attention mechanisms in transformers (Ramsauer et al., 2020; Vaswani et al., 2017). Variants of modern Hopfield networks were then proposed to both achieve higher capacity and recover different types of attention mechanisms (Hu et al., 2023; Santos et al., 2025; Burns & Fukai, 2023; Krotov, 2023; Wu et al., 2024).

Neural Population Geometry. In computational neuroscience, the geometric aspects of neural population activities have gained growing attention in recent years (Kriegeskorte & Wei, 2021). Most works study the activity patterns of animals when they encounter different stimuli. For example, Chapman & Störmer (2024) studies population geometry of the visual system to analyze which visual features animals attend to. Panzeri et al. (2022) studies how neural populations represent and process information by analyzing the activity correlations between place cells in the hippocampus. Besides studying the structure of neural representation spaces, researchers also try to understand the intrinsic geometry in those spaces. Specifically, growing evidence shows that hyperbolic geometry naturally occurs in various biological systems (Kagel & Sharpee, 2025; Lecca et al., 2023; Sharpee, 2019; Lee et al., 2024; Allard & Serrano, 2020). Zhang et al. (2023) shows that the semi-metric between hippocampal place cells has the same topological structure as hyperbolic geometry, suggesting a hyperbolic cognitive map in animal brains for spatial information. Next, they show that when animals gain experience, the radius of the latent cognitive map grows larger. This finding provides a unique angle to connect learning to/with neural population geometry. Similarly, Zhou et al. (2018); Ghaninia et al. (2022) find that besides spatial information, animals also encode odors into a latent hyperbolic map using a similar analytical tool used in (Zhang et al., 2023). There are two major differences between our work and (Zhang et al., 2023; Zhou et al., 2018; Ghaninia et al., 2022). First, our results are mainly theoretical, whereas previous works obtain their results empirically using topological data analysis. Next, their studies focused on showing that neuron-to-neuron correlations are topologically hyperbolic, hereas we instead show that inter-stimuli distances are hyperbolic from a metric perspective.

Methods for Geometric Analysis of Neural Representations. Various algorithms have been proposed to (1) measure the underlying geometry of neural representations and (2) compare the similarities between two sets of neural responses. For (1), a typical example is Zhang et al. (2023), where they utilize a technique in topological data analysis (TDA) called the Betti curve. In topology, the Betti numbers are used to distinguish topological spaces based on the number of n-dimensional holes in the space. In Zhang et al. (2023), the authors compute the pair-wise dissimilarity between neurons, and construct a graph with vertices being the neurons. The vertices are connected if their pair-wise dissimilarity is below some threshold τ. The Betti number is computed for each τ, which forms the Betti curve for each Betti number. This method allows researchers to compare experimental data to well-defined topological spaces.

D. Additional Background

D.1. Visualization of δ-thin Triangles

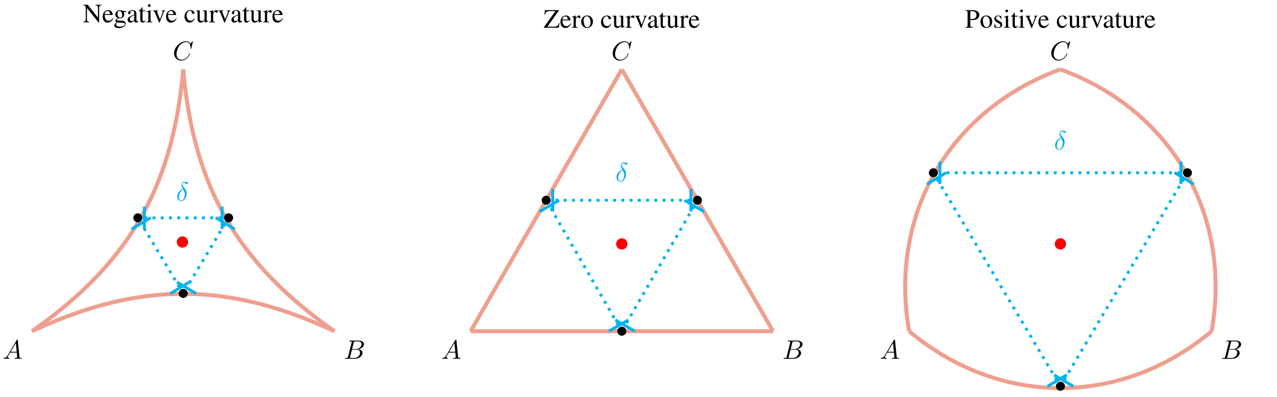

Figure 3. **δ-thin triangles:** the δ-thin triangles refer to the geodesic triangles formed by points A, B, C connected with orange geodesics. For each triangle, the red dot denotes its center, black dots refer to the midpoints of its sides, cyan dot lines measures the length between any two midpoints. Hyperbolic spaces exhibit uniformly thin triangles (small δ), Euclidean spaces are borderline, and spherical/positively curved spaces exhibit "fatter" triangles (larger δ). The arrows indicate representative distances from midpoints of each side to the center of the circle. The diameter of this set of midpoints is bounded by δ, and the circle's radius gives $\frac{\delta}{2}$. on one side to the union of the other two sides, all of which are uniformly bounded by δ. The figure is schematic and not drawn to scale.

D.2. Properties of Hyperbolic Geometry

Lemma D.1. *Let $\kappa < 0$, $\alpha := \sqrt{|\kappa|}$, and let*

$$\boldsymbol{x} = \mathrm{Exp}_o(a), \qquad \boldsymbol{y} = \mathrm{Exp}_o(b), \qquad \boldsymbol{z} = \mathrm{Exp}_o(c)$$

be hyperboloid embeddings of $a, b, c \in T_o\mathcal{M} \simeq \mathbb{R}^d$. Define the squared Euclidean distance gap

$$\Delta^{\mathrm{E}}(a; b, c) \;:=\; \|a - c\|_2^2 - \|a - b\|_2^2,$$

and let

$$g(r) := \frac{\sinh(\alpha r)}{r}, \qquad r > 0,$$

with $g(0) = \alpha$.

Then the Lorentz inner-product difference admits the exact decomposition

$$\langle \boldsymbol{x}, \boldsymbol{y} \rangle_L - \langle \boldsymbol{x}, \boldsymbol{z} \rangle_L = \frac{1}{|\kappa|}\, g(\|a\|_2)\, \frac{g(\|b\|_2)}{2}\, \Delta^{\mathrm{E}}(a; b, c) \;-\; \mathsf{Pen}_\kappa(a; b, c), \tag{D.1}$$

where the penalty term $\mathsf{Pen}_\kappa(a; b, c)$ is given by

$$\mathsf{Pen}_\kappa(a; b, c) := \frac{1}{|\kappa|} \cosh(\alpha\|a\|_2)\Big(\cosh(\alpha\|b\|_2) - \cosh(\alpha\|c\|_2)\Big) \tag{D.2}$$

$$+ \frac{1}{|\kappa|}\, g(\|a\|_2) \left[\frac{g(\|b\|_2)}{2} \big(\|c\|_2^2 - \|b\|_2^2\big) - \big(g(\|b\|_2) - g(\|c\|_2)\big) \langle a, c \rangle \right]. \tag{D.3}$$

Moreover, if $\|a\|_2, \|b\|_2, \|c\|_2 \in [r_{\min}, r_{\max}]$ with $0 < r_{\min} \le r_{\max} < \infty$, then

$$\langle \boldsymbol{x}, \boldsymbol{y} \rangle_L - \langle \boldsymbol{x}, \boldsymbol{z} \rangle_L \;\ge\; A_\kappa(r_{\min}; a)\, \Delta^{\mathrm{E}}(a; b, c) \;-\; \mathsf{Pen}_\kappa(r_{\min}, r_{\max}), \tag{D.4}$$

where

$$A_\kappa(r_{\min}; a) \;:=\; \frac{1}{|\kappa|}\, g(\|a\|_2)\, \frac{g(r_{\min})}{2},$$

and the uniform penalty bound

$$\mathsf{Pen}_\kappa(r_{\min}, r_{\max}) \;:=\; \sup_{\|a\|_2, \|b\|_2, \|c\|_2 \in [r_{\min}, r_{\max}]} \mathsf{Pen}_\kappa(a; b, c)$$

is finite and satisfies the rate

$$\mathsf{Pen}_\kappa(r_{\min}, r_{\max}) = \mathcal{O}\left(\frac{1}{|\kappa|}\, e^{2\alpha r_{\max}}\, r_{\max}^2\right), \qquad \alpha = \sqrt{|\kappa|},$$

with constants depending only on $r_{\min} > 0$.

Proof of Lemma D.1. We rewrite the Lorentz inner-product difference in terms of the *squared Euclidean distance gap*

$$\Delta^{\mathrm{E}}(a; b, c) \;:=\; \|a - c\|_2^2 - \|a - b\|_2^2.$$

Let $\alpha := \sqrt{|\kappa|}$ and $s^2 = 1/|\kappa|$. Under the hyperboloid model, for any $u \in T_o\mathcal{M} \simeq \mathbb{R}^d$,

$$\mathrm{Exp}_{\boldsymbol{o}}(u) = \Big(s \cosh(\alpha\|u\|_2),\; s\, \frac{\sinh(\alpha\|u\|_2)}{\|u\|_2}\, u\Big).$$

Define

$$\boldsymbol{x} := \mathrm{Exp}_{\boldsymbol{o}}(a), \qquad \boldsymbol{y} := \mathrm{Exp}_{\boldsymbol{o}}(b), \qquad \boldsymbol{z} := \mathrm{Exp}_{\boldsymbol{o}}(c),$$

and for convenience set

$$g(r) := \frac{\sinh(\alpha r)}{r}, \qquad r > 0,$$

(with $g(0) = \alpha$). A direct substitution gives the exact identity

$$\langle \boldsymbol{x}, \boldsymbol{y}\rangle_L - \langle \boldsymbol{x}, \boldsymbol{z}\rangle_L = -\frac{1}{|\kappa|} \cosh(\alpha\|a\|_2)\Big(\cosh(\alpha\|b\|_2) - \cosh(\alpha\|c\|_2)\Big) \tag{D.5}$$

$$+ \frac{1}{|\kappa|} g(\|a\|_2)\Big(g(\|b\|_2)\,\langle a, b\rangle - g(\|c\|_2)\,\langle a, c\rangle\Big). \tag{D.6}$$

By expanding squared norms,

$$\begin{aligned}\Delta^{\mathrm{E}}(a; b, c) &= \big(\|a\|_2^2 + \|c\|_2^2 - 2\langle a, c\rangle\big) - \big(\|a\|_2^2 + \|b\|_2^2 - 2\langle a, b\rangle\big)\\ &= 2\big(\langle a, b\rangle - \langle a, c\rangle\big) + \big(\|c\|_2^2 - \|b\|_2^2\big).\end{aligned}$$

Hence

$$\langle a, b\rangle - \langle a, c\rangle = \frac{1}{2}\Delta^{\mathrm{E}}(a; b, c) - \frac{1}{2}\big(\|c\|_2^2 - \|b\|_2^2\big). \tag{D.7}$$

Rewrite the bracket in (D.6) as

$$g(\|b\|_2)\,\langle a, b\rangle - g(\|c\|_2)\,\langle a, c\rangle = g(\|b\|_2)\big(\langle a, b\rangle - \langle a, c\rangle\big) + \big(g(\|b\|_2) - g(\|c\|_2)\big)\,\langle a, c\rangle\,.$$

Substituting (D.7) yields

$$g(\|b\|_2)\,\langle a, b\rangle - g(\|c\|_2)\,\langle a, c\rangle = \frac{g(\|b\|_2)}{2}\,\Delta^{\mathrm{E}}(a; b, c) - \frac{g(\|b\|_2)}{2}\big(\|c\|_2^2 - \|b\|_2^2\big) + \big(g(\|b\|_2) - g(\|c\|_2)\big)\,\langle a, c\rangle\,. \tag{D.8}$$

Plugging (D.8) into (D.6) and combining with (D.5) gives the *exact decomposition*

$$\langle \boldsymbol{x}, \boldsymbol{y}\rangle_L - \langle \boldsymbol{x}, \boldsymbol{z}\rangle_L = \underbrace{\frac{1}{|\kappa|}\, g(\|a\|_2)\,\frac{g(\|b\|_2)}{2}\,\Delta^{\mathrm{E}}(a; b, c)}_{\text{main term}} \;-\; \underbrace{\mathsf{Pen}_\kappa(a; b, c)}_{\text{penalty}}, \tag{D.9}$$

where the penalty $\mathsf{Pen}_\kappa(a;b,c)$ is given explicitly by

$$\mathsf{Pen}_\kappa(a;b,c) := \frac{1}{|\kappa|}\cosh(\alpha\|a\|_2)\Big(\cosh(\alpha\|b\|_2) - \cosh(\alpha\|c\|_2)\Big) \tag{D.10}$$

$$+\frac{1}{|\kappa|}\,g(\|a\|_2)\left[\frac{g(\|b\|_2)}{2}\big(\|c\|_2^2 - \|b\|_2^2\big) - \big(g(\|b\|_2) - g(\|c\|_2)\big)\,\langle a, c\rangle\right]. \tag{D.11}$$

Equations (D.9)–(D.11) are obtained from the closed-form hyperboloid exponential map.

Assume $\|a\|_2, \|b\|_2, \|c\|_2 \in [r_{\min}, r_{\max}]$ with $0 < r_{\min} \leq r_{\max} < \infty$. Since $g(\cdot)$ is increasing on $(0,\infty)$, we have $g(\|b\|_2) \geq g(r_{\min})$, and thus the main term in (D.9) admits the uniform lower bound

$$\frac{1}{|\kappa|}g(\|a\|_2)\frac{g(\|b\|_2)}{2}\,\Delta^{\mathrm{E}}(a;b,c) \;\geq\; \frac{1}{|\kappa|}g(\|a\|_2)\frac{g(r_{\min})}{2}\,\Delta^{\mathrm{E}}(a;b,c).$$

Define the amplification factor

$$A_\kappa(r_{\min};a) \;:=\; \frac{1}{|\kappa|}g(\|a\|_2)\frac{g(r_{\min})}{2}.$$

Then (D.9) implies the deterministic inequality

$$\langle \boldsymbol{x}, \boldsymbol{y}\rangle_L - \langle \boldsymbol{x}, \boldsymbol{z}\rangle_L \;\geq\; A_\kappa(r_{\min};a)\,\Delta^{\mathrm{E}}(a;b,c) \;-\; \sup_{\|a\|_2,\|b\|_2,\|c\|_2\in[r_{\min},r_{\max}]} \mathsf{Pen}_\kappa(a;b,c). \tag{D.12}$$

Finally, note that the supremum penalty term in (D.12) is finite for fixed $(r_{\min}, r_{\max}, \kappa)$ and can be bounded explicitly using $\cosh(\alpha r) \lesssim e^{\alpha r}$ and $g(r) = \sinh(\alpha r)/r \lesssim e^{\alpha r}/r$ on $[r_{\min}, r_{\max}]$. In particular, one obtains the coarse rate

$$\sup \mathsf{Pen}_\kappa(a;b,c) = \mathcal{O}\left(\frac{1}{|\kappa|}e^{2\alpha r_{\max}}\,r_{\max}^2\right), \qquad \alpha = \sqrt{|\kappa|},$$

where the hidden constant depends only on $r_{\min} > 0$ (through $1/r_{\min}$ factors in g and its Lipschitz constant on the interval). □

Lemma D.2. *Let $\kappa < 0$, $\alpha = \sqrt{|\kappa|}$, and $\boldsymbol{v} = \mathrm{Exp}_{\boldsymbol{o}}(\mathtt{v})$ with $\mathtt{v} \in \mathbb{R}^d$. Let $\boldsymbol{\xi}_\rho = \mathrm{Exp}_{\boldsymbol{o}}(s_\rho)$ for all $\rho \in [M]$. Fix $\mu \neq \nu$ and define the squared-distance gap*

$$\Delta^{\mathrm{E}}_{\mu\nu}(\mathtt{v}) := \|\mathtt{v} - s_\nu\|_2^2 - \|\mathtt{v} - s_\mu\|_2^2.$$

Assume $\|\mathtt{v}\|_2, \|s_\mu\|_2, \|s_\nu\|_2 \in [r_{\min}, r_{\max}]$. Then

$$\Delta^{\mathrm{H}}_{\mu\nu}(\boldsymbol{v}) = \langle \boldsymbol{v}, \boldsymbol{\xi}_\mu\rangle_L - \langle \boldsymbol{v}, \boldsymbol{\xi}_\nu\rangle_L \;\geq\; f_\kappa(r_{\min})\,\Delta^{\mathrm{E}}_{\mu\nu}(\mathtt{v}) \;-\; \tilde{\mathsf{Pen}}_\kappa(r_{\min}, r_{\max}),$$

where

$$f_\kappa(r) := \frac{1}{2|\kappa|}\left(\frac{\sinh(\alpha r)}{r}\right)^2,$$

and $\tilde{\mathtt{Pen}}_\kappa(r_{\min}, r_{\max})$ is the uniform penalty bound from Corollary Theorem D.3.

Proof. Apply Lemma D.1 with $a = \mathtt{v}$, $b = s_\mu$, $c = s_\nu$ and $(x,y,z) = (\boldsymbol{v}, \boldsymbol{\xi}_\mu, \boldsymbol{\xi}_\nu)$. Then use the uniformization on $[r_{\min}, r_{\max}]$ and the definition of f_κ. □

Corollary D.3. *Let $\kappa < 0$, $\alpha := \sqrt{|\kappa|}$, and assume the stored patterns satisfy $r_{\min} \leq \|s_\mu\|_2 \leq r_{\max}$ for all μ, with $\Delta r := r_{\max} - r_{\min}$. Define*

$$\Delta^{\mathrm{E}}_\mu := \min_{\nu\neq\mu}\|s_\mu - s_\nu\|_2^2, \qquad \Delta^{\mathrm{H}}_\mu := \min_{\nu\neq\mu}\Big(\langle \xi_\mu, \xi_\mu\rangle_L - \langle \xi_\mu, \xi_\nu\rangle_L\Big).$$

Then for each μ,

$$\Delta^{\mathrm{H}}_\mu \;\geq\; f_\kappa(r_{\min})\,\Delta^{\mathrm{E}}_\mu \;-\; \tilde{\mathtt{Pen}}_\kappa(r_{\min}, r_{\max}),$$

where $f_\kappa(r) = \frac{1}{2|\kappa|}\big(\frac{\sinh(\alpha r)}{r}\big)^2$*, and the penalty admits the bound*

$$\widetilde{\mathsf{Pen}}_\kappa(r_{\min}, r_{\max}) \;\le\; \frac{1}{|\kappa|}\Big[\alpha\,\cosh^2(\alpha r_{\max})\,\Delta r \;+\; C_g(r_{\min}, r_{\max})\,g(r_{\max})\,r_{\max}^2\,\Delta r\Big],$$

with $g(r) = \sinh(\alpha r)/r$ *and*

$$C_g(r_{\min}, r_{\max}) \;\coloneqq\; \sup_{r\in[r_{\min}, r_{\max}]} \big|g'(r)\big|.$$

In particular, since $\cosh(\alpha r) \lesssim e^{\alpha r}$ *and* $g(r) \lesssim e^{\alpha r}/r$ *on* $[r_{\min}, r_{\max}]$*, we also have the following rate*

$$\widetilde{\mathsf{Pen}}_\kappa(r_{\min}, r_{\max}) = \mathcal{O}\left(\frac{1}{|\kappa|}\,e^{2\alpha r_{\max}}\,r_{\max}^2\,\Delta r\right), \qquad \Delta r = r_{\max} - r_{\min}.$$

D.3. Technical Tools

Definition D.4. For a point process $\boldsymbol{X}$, its intensity measure ν, evaluated on a Borel set B, is defined as

$$\nu(B) = \mathbb{E}[\boldsymbol{X}(B)].$$

Theorem D.5 (Campbell's Theorem (Baddeley et al., 2007))**.** *Let $\boldsymbol{X}$ be a point process on S and let $f : S \to \mathbb{R}$ be a measurable function. Then the random sum*

$$T \coloneqq \sum_{x \in \boldsymbol{X}} f(x)$$

is a random variable, with expected value

$$\mathbb{E}\left[\sum_{x \in \boldsymbol{X}} f(x)\right] = \int_S f(x)\, \nu(dx).$$

Further, if $\boldsymbol{X}$ is a point process on $\mathbb{R}^d$ with constant intensity ρ, the expectation becomes

$$\mathbb{E}\left[\sum_{x \in \boldsymbol{X}} f(x)\right] = \rho \int_{\mathbb{R}^d} f(x)\, dx,$$

where $\nu(dx) = \rho dx$.

Lemma D.6 (Markov's Inequality)**.** *Let X be a nonnegative random variable and $a > 0$. Then*

$$\Pr[X \geq a] \leq \frac{\mathbb{E}[X]}{a}.$$

E. Proof of Main Text Results

E.1. Proof of Proposition 2.2

Proof. The objective is convex in z. Differentiating and setting to zero:

$$\frac{d}{dz}\,\mathbb{E}_{p(\mu|v)}\left[\|\xi_\mu - z\|_2^2\right] = -2\sum_{\mu=1}^{M} p(\mu \mid v)\,(\xi_\mu - z) = 0. \tag{E.1}$$

Solving directly:

$$z^* = \sum_{\mu=1}^{M} p(\mu \mid v)\,\xi_\mu. \tag{E.2}$$

Substituting the Boltzmann posterior:

$$z^* = \sum_{\mu=1}^{M} \frac{e^{\langle v,\,\xi_\mu\rangle}}{\sum_\nu e^{\langle v,\,\xi_\nu\rangle}}\,\xi_\mu = \sum_{\mu=1}^{M} \mathrm{softmax}_\mu\big(h_1^{\mathrm{MHN}}(v),\ldots,h_M^{\mathrm{MHN}}(v)\big)\,\xi_\mu = \mathrm{MHN}(v). \tag{E.3}$$

□

E.2. Proof of Theorem 4.2

Define the kernel

$$K(s,s') \coloneqq \sum_{i=1}^{N} \lambda_i(s)\lambda_i(s'), \quad \lambda_i(s) = \exp\left(-\frac{||s-s_i||_2^2}{2\sigma_i^2}\right).$$

Define the corresponding metric-like distance

$$d(s,s') = -\ln\left(K(s,s')\right).$$

Our goal is to show that for any 4 points (s_a, s_b, s_c, s_d), the quantity

$$\Delta = d(s_a,s_b) + d(s_c,s_d) - \max\{d(s_a,s_c)+d(s_b,s_d), d(s_a,s_d)+d(s_b,s_c)\},$$

is bounded by a constant 2δ with high probability.

Definition E.1.

$$K_{\max}(s,s') \coloneqq \max_i \left\langle \lambda_i(s), \lambda_i(s')\right\rangle, \quad d^*(s,s') \coloneqq -\ln(K_{\max}(s,s')).$$

Definition E.2. Let $i \coloneqq \arg\max_{j\in[N]} \sigma_j$, then

$$d_i(s,s') \coloneqq -\ln(\lambda_i(s)\lambda_i(s')).$$

Lemma E.3. *For any $j \in [N]$, d_j is 0-hyperbolic.*

Proof. Observe

$$\begin{aligned} d_j(s,s') &= -\ln\big(\lambda_j(s)\lambda_j(s')\big) \\ &= \frac{1}{2\sigma_j^2}(||s-s_j||_2^2 + ||s'-s_j||_2^2) \\ &= a_j(w_j(s)+w_j(s')), \end{aligned}$$

where $a_j = \frac{1}{2\sigma_j^2}$, and $w_j(s) = ||s-s_j||_2^2$.

The proof concludes by direct calculation of the 4-point condition. □

Proof of Theorem 4.2.

Let $i = \arg\max_{j\in[N]} \sigma_j$, and decompose

$$K(s,s') = \lambda_i(s)\lambda_i(s') + R_i(s,s'), \qquad R_i(s,s') \coloneqq K(s,s') - \lambda_i(s)\lambda_i(s') = \sum_{j\neq i} \lambda_j(s)\lambda_j(s') \geq 0.$$

Then

$$\begin{aligned} d(s,s') &= -\ln\left(\lambda_i(s)\lambda_i(s')\left(1+\frac{R_i(s,s')}{\lambda_i(s)\lambda_i(s')}\right)\right) \\ &= d_i(s,s') - \varepsilon(s,s'), \end{aligned}$$

where $\varepsilon(s,s') \coloneqq \ln\Big(1+\frac{R_i(s,s')}{\lambda_i(s)\lambda_i(s')}\Big) \geq 0$.

For four points (s_x, s_y, s_z, s_w), define the three pair-sums under d and d_i:

$$S_k = (k\text{-th pair-sum under } d), \qquad S_k^i = (k\text{-th pair-sum under } d_i), \qquad k = 1,2,3.$$

For example, $S_1 = d(x,y) + d(z,w)$ and $S_1^i = d_i(x,y) + d_i(z,w)$. By the decomposition,

$$S_k = S_k^i - P_k, \quad \text{where } P_k \coloneqq \sum_{\text{two pairs in } S_k} \varepsilon(\cdot,\cdot), \quad P_k \in [0,\, 2\varepsilon_{\max}],$$

and $\varepsilon_{\max} \coloneqq \max_{(u,v)\in\binom{(x,y,z,w)}{2}} \varepsilon(u,v)$.

Claim: $\Delta(x,y,z,w) \leq 2\varepsilon_{\max}$.

Proof of Claim. By Lemma E.3, $S_1^i = S_2^i = S_3^i \coloneqq S$. Therefore $S_k = S - P_k$, and

$$\Delta = \max_{(1)} S_k - \max_{(2)} S_k = \min_{(1)} P_k - \min_{(2)} P_k \le \max_k P_k - \min_k P_k \le 2\varepsilon_{\max},$$

where the order flips because S_k is decreasing in P_k. □

Note that now the goal is to check the condition where

$$\varepsilon(s, s') = \ln\left(1 + \frac{R_i(s, s')}{\lambda_i(s)\lambda_i(s')}\right) < \delta,$$

for some constant $\delta > 0$.

Let the centers $\{s_j\}_{j \neq i}$ be distributed as a Poisson point process $\Phi \sim \mathrm{PPP}(\rho)$ on $[0, L]^D$ with intensity $\rho = \frac{N}{L^D}$. By the parallelogram identity, for any center s_j:

$$||s - s_j||_2^2 + ||s' - s_j||_2^2 = 2\,||m - s_j||_2^2 + \frac{1}{2}\,||s - s'||_2^2\,,$$

where $m = \frac{1}{2}(s + s')$ is the midpoint. Substituting into the product of activations,

$$\lambda_j(s)\lambda_j(s') = \exp\left(-\frac{||s - s_j||_2^2 + ||s' - s_j||_2^2}{2\sigma_j^2}\right) = \underbrace{\exp\left(-\frac{||s - s'||_2^2}{4\sigma_j^2}\right)}_{\le 1} \cdot \exp\left(-\frac{||m - s_j||_2^2}{\sigma_j^2}\right).$$

By Campbell's theorem (Theorem D.5), the expected residual satisfies

$$\begin{aligned}
\mathbb{E}[R_i \mid \{\sigma_j\}] &= \mathbb{E}\left[\sum_{s_j \in \Phi} \lambda_j(s)\lambda_j(s')\right] \\
&\le \rho \int_{\mathcal{S}} \exp\left(-\frac{||m - s_j||_2^2}{\sigma_j^2}\right) ds_j \\
&\le \rho \int_{\mathcal{S}} \exp\left(-\frac{||u||_2^2}{\sigma_j^2}\right) du \\
&= \rho(\pi\sigma_j^2)^{\frac{D}{2}}\,,
\end{aligned}$$

where the second inequality extends the domain to $\mathbb{R}^D$ (after the change of variable $u = m - s_j$), and the final equality applies the standard Gaussian integral.

Finally,

$$\mathbb{E}_{s_j}[\lambda_j(s)\lambda_j(s') \mid \sigma_j] \le \frac{1}{L^D} \int_{\mathbb{R}^D} e^{-||m - s_j||_2^2/\sigma_j^2} ds_j = \frac{(\pi\sigma_j^2)^{\frac{D}{2}}}{L^D},$$

where m is the midpoint, i.e. $m = \frac{1}{2}(s + s')$, and the second equation comes from the closed-form of the Gaussian product integral.

Summing over $j \neq i$ gives

$$\mathbb{E}[R_i] \le (N - 1)\frac{(\pi)^{D/2}\mathbb{E}[\sigma_j^D]}{L^D} = C_D \cdot \mu,$$

where $C_D = \pi^{D/2}$, and $\mu = N\mathbb{E}[\sigma_j^D]/L^D$.

Define the event

$$\mathcal{A} \coloneqq \{\sigma_i \ge cL\}, \quad c \in (0, 1).$$

By $||s - s_i|| \leq L\sqrt{D}$, on $\mathcal{A}$:

$$\lambda_i(s)\lambda_i(s') \geq e^{-\frac{D}{c^2}}. \tag{E.4}$$

By Markov's inequality (Lemma D.6):

$$\Pr[\varepsilon_{\max} \geq 2\delta \mid \mathcal{A}] \leq \frac{\mathbb{E}[\varepsilon_{\max} \mid \mathcal{A}]}{2\delta} \leq \frac{6e^{\frac{D}{c^2}} C_D \mu}{2\delta}. \tag{E.5}$$

The probability that $\mathcal{A}$ fails is given by the CDF of the exponential distribution:

$$\Pr\left[\sigma_i < cL\right] = \left(1 - e^{-\frac{cL}{\beta}}\right)^N \leq \exp\left(-Ne^{\frac{-cL}{\beta}}\right),$$

where the inequality comes from $(1-x)^N \leq e^{-Nx}$, for $x \in [0,1]$.

Setting the Markov bound in (E.5) equal to a target failure probability $\eta \in (0,1)$, we obtain $2\delta = \frac{6\,e^{D/c^2} C_D \mu}{\eta}$. Finally, by a union bound over $\{\varepsilon_{\max} \geq 2\delta \mid \mathcal{A}\}$ and $\mathcal{A}^c$, with probability at least $1 - \eta - \Pr[\mathcal{A}^c]$ we have

$$\Delta \leq 2\varepsilon_{\max} \leq 2\delta = \frac{6\,e^{\frac{D}{c^2}} C_D \mu}{\eta}. \tag{E.6}$$

□

E.2.1. Breaking Conditions

Distributions of σ. Here we study the worst-case 4-point condition, where all 4 points sit at corners of the domain. Assume the four points (a, b, c, d) form a square of side L in $\mathbb{R}^D$.

We can compute the δ explicitly as follows.

The diagonals between opposite pairs have Euclidean distance $L\sqrt{2}$, and the kernel distance $d(s, s') \approx \frac{(L\sqrt{2})^2}{4\mathbb{E}[\sigma^2]} = \frac{2L^2}{4\mathbb{E}[\sigma^2]}$ (ignoring the constant $\ln N$). The sum of diagonals is $S_1 = \frac{4L^2}{4\mathbb{E}[\sigma^2]}$. The sides between adjacent pairs have Euclidean distance L, and the kernel distance $d(s, s') \approx \frac{L^2}{4\mathbb{E}[\sigma^2]}$. The sum of sides is $S_2 = \frac{2L^2}{4\mathbb{E}[\sigma^2]}$.

δ is then calculated as

$$\delta = S_1 - S_2 = \frac{L^2}{2\mathbb{E}[\sigma^2]}. \tag{E.7}$$

From (E.7) we see that any bounded distributions, such as uniform or constant-size place fields, do not preserve constant δ as $L \to \infty$.

Further, plugging other distributions into the proof of Theorem 4.2 shows that the log-normal distribution, whose tail decays more slowly than a Gaussian, also yields $\delta \to 0$. This implies that the tail behavior of the distribution affects the hyperbolicity of neural activities. ok

Other Breaking Conditions. Here we discuss conditions of other parameters in the system that would break statistical hyperbolicity. Specifically, breaking hyperbolicity means Δ is increasing in L. Based on Equation (E.6), one straightforward way to break hyperbolicity is to have

$$N = o(\frac{\mathbb{E}[\sigma^D]}{L^D}).$$

Here we interpret this as the number of place fields instead of the number of active place cells for two reasons. First, we suspect that, in the multi-field case, with each neuron having $K > 0$ place fields, the hyperbolicity of the space is controlled by the variable NK, which is required to scale linearly w.r.t. the size of the environment. This aligns with the experimental findings in (Harland et al., 2021). Second, ideally and theoretically, the hyperbolicity of a metric space should not depend on the dimensionality of the space, but the curvature of it, which is more closely related to how place fields cover the

environment. However, this also shows a limitation of our work, where the number of active neurons and the number of place fields are coupled. Notably, while having the constant $c \geq 1$ does not necessarily break the hyperbolicity, it is not biologically plausible to have place fields larger than the size of the environment.

E.3. Proof of Theorem 4.8

We organize the proof as follows. In Section E.4, we state two geometric facts derived from Rauch's comparison theorem, together with the hyperbolic margin event and a Euclidean Chernoff condition on the query noise. In Section E.5, we establish a deterministic decoder bound that controls the recall error under the margin event. In Section E.6, we bridge the Euclidean and hyperbolic margins through Lemma D.2 and derive a sub-Gaussian Chernoff bound on the hyperbolic margin event. Section E.7 combines these ingredients to yield the asymptotic capacity bound, and Section E.8 isolates several regimes of interest.

E.4. Preliminaries

Throughout this section we work on the hyperboloid model $\mathbb{H}^d_\kappa$ with curvature $\kappa < 0$, and write $\alpha := \sqrt{|\kappa|}$. We denote by $\mathbf{o}$ the origin of $\mathbb{H}^d_\kappa$, by $\mathrm{Exp}_p(\cdot)$ the exponential map at $p \in \mathbb{H}^d_\kappa$, and by $d_g(\cdot,\cdot)$ the geodesic distance. The stored patterns are $\boldsymbol{\xi}_\rho = \mathrm{Exp}_{\mathbf{o}}(x_\rho)$ for $\rho \in [M]$, with anchor points $x_\rho \in \mathbb{R}^d$, and the query is $\mathbf{v} = \mathrm{Exp}_{\mathbf{o}}(\mathrm{v})$ with $\mathrm{v} = x_\mu + \boldsymbol{\sigma} z$, where $z \sim \mathcal{N}(0, I_d)$ and $\mu \in [M]$ is the planted index.

We rely on two standard consequences of Rauch's comparison theorem (Rauch, 1951) on the simply-connected complete Hadamard manifold $\mathbb{H}^d_\kappa$.

(L1) *The inverse exponential map is globally* 1*-Lipschitz*: for any $p, x, y \in \mathbb{H}^d_\kappa$,

$$\left\|\mathrm{Exp}_p^{-1}(x) - \mathrm{Exp}_p^{-1}(y)\right\|_p \;\le\; d_g(x,y).$$

In particular, $\|\,\mathrm{Exp}_p^{-1}(x)\|_p = d_g(p,x)$.

(L2) *The exponential map is locally Lipschitz with explicit constant*: for any $u, w \in B(0,R) \subset T_p\mathbb{H}^d_\kappa$,

$$d_g\big(\mathrm{Exp}_p(u), \mathrm{Exp}_p(w)\big) \;\le\; L_{\mathrm{Exp}}(R)\, \|u-w\|_p, \qquad L_{\mathrm{Exp}}(R) := \frac{\sinh(\alpha R)}{\alpha R}.$$

We next introduce the central random object of our analysis.

Definition E.4 (Hyperbolic margin event)**.** For a query $\mathbf{v} \in \mathbb{H}^d_\kappa$ and indices $\mu \neq \nu$, the *hyperbolic gap* is

$$\Delta^{\mathrm{H}}_{\mu\nu}(\mathbf{v}) \;:=\; \langle \mathbf{v}, \boldsymbol{\xi}_\mu\rangle_{\mathscr{L}} - \langle \mathbf{v}, \boldsymbol{\xi}_\nu\rangle_{\mathscr{L}}.$$

For $\Gamma > 0$, the *margin event* at planted index μ is

$$\mathscr{M}^{\mathrm{H}}_\mu(\Gamma) \;:=\; \Big\{\min_{\nu\neq\mu} \Delta^{\mathrm{H}}_{\mu\nu}(\mathbf{v}) \ge \Gamma\Big\}. \tag{E.8}$$

The high-level strategy is to ensure that, on $\mathscr{M}^{\mathrm{H}}_\mu(\Gamma)$ with Γ sufficiently large, the softmax weights concentrate on the planted index μ, and that this margin event itself holds with high probability under the following Euclidean concentration assumption on the query noise.

Assumption E.5 (Euclidean Chernoff condition)**.** Fix distinct $\mu \neq \nu$, and under the conditional law $\mathrm{v} \mid \mu$ define

$$\Delta^{\mathrm{E}}_{\mu\nu}(\mathrm{v}) \;:=\; \|\mathrm{v} - x_\nu\|_2^2 - \|\mathrm{v} - x_\mu\|_2^2.$$

There exist constants $\gamma_{\mathrm{E}} > 0$, $K_{\mathrm{E}} < \infty$ and $d_0 \in \mathbb{N}$ such that for all $d \ge d_0$:

(A1) $\mathbb{E}[\Delta^{\mathrm{E}}_{\mu\nu}(\mathrm{v}) \mid \mu] \ge \gamma^{\mathrm{E}} d$;

(A2) conditioned on μ, the centered gap $\Delta^{\mathrm{E}}_{\mu\nu}(\mathrm{v}) - \mathbb{E}[\Delta^{\mathrm{E}}_{\mu\nu}(\mathrm{v}) \mid \mu]$ is sub-Gaussian with proxy variance at most $K_{\mathrm{E}}\boldsymbol{\sigma}^2 d$.

E.5. Error Under Margin Event

We first show that conditional on the margin event $\mathscr{M}_\mu^{\mathrm{H}}(\Gamma)$, the softmax weights concentrate sharply on the planted index.

Lemma E.6 (Softmax concentration). *On $\mathscr{M}_\mu^{\mathrm{H}}(\Gamma)$,*

$$\sum_{\nu\neq\mu} w_\nu(\mathbf{v}) \;\leq\; (M-1)\, e^{-\Gamma}, \qquad w_\mu(\mathbf{v}) \;\geq\; \frac{1}{1+(M-1)\, e^{-\Gamma}}. \tag{E.9}$$

Proof. For each $\nu \neq \mu$, $w_\nu(\mathbf{v})/w_\mu(\mathbf{v}) = \exp\bigl(-\Delta^{\mathrm{H}}_{\mu\nu}(\mathbf{v})\bigr) \leq e^{-\Gamma}$ on $\mathscr{M}_\mu^{\mathrm{H}}(\Gamma)$. Summing over $\nu \neq \mu$ and using $\sum_\rho w_\rho(\mathbf{v}) = 1$ yields both inequalities. □

We now translate softmax concentration into a deterministic recall guarantee.

Proposition E.7 (Decoder bound). *Set $u_\rho := \mathrm{Exp}_{\mathbf{v}}^{-1}(\boldsymbol{\xi}_\rho) \in T_{\mathbf{v}}\mathbb{H}^d_\kappa$ and $\bar{u} := \sum_\rho w_\rho(\mathbf{v})\, u_\rho$, so that $H(\mathbf{v}) = \mathrm{Exp}_{\mathbf{v}}(\bar{u})$. Define $R(\mathbf{v}) := \max_\rho \|u_\rho\|_{\mathbf{v}} = \max_\rho d_g(\mathbf{v}, \boldsymbol{\xi}_\rho)$, where the second equality follows from* (L1). *Then on $\mathscr{M}_\mu^{\mathrm{H}}(\Gamma)$,*

$$d_g\bigl(H(\mathbf{v}), \boldsymbol{\xi}_\mu\bigr) \;\leq\; 2\, r_{\max}\, L_{\mathrm{Exp}}\bigl(R(\mathbf{v})\bigr)\, (M-1)\, e^{-\Gamma}. \tag{E.10}$$

Proof. By (L2) applied with $u = \bar{u}$ and $w = u_\mu \in B(0, R(\mathbf{v}))$,

$$d_g\bigl(H(\mathbf{v}), \boldsymbol{\xi}_\mu\bigr) \;=\; d_g\bigl(\mathrm{Exp}_{\mathbf{v}}(\bar{u}), \mathrm{Exp}_{\mathbf{v}}(u_\mu)\bigr) \;\leq\; L_{\mathrm{Exp}}\bigl(R(\mathbf{v})\bigr)\, \|\bar{u} - u_\mu\|_{\mathbf{v}}. \tag{E.11}$$

By the triangle inequality and (L1) applied to each pair $(\boldsymbol{\xi}_\nu, \boldsymbol{\xi}_\mu)$,

$$\|\bar{u} - u_\mu\|_{\mathbf{v}} \;=\; \Bigl\|\sum_{\nu\neq\mu} w_\nu(\mathbf{v})\,(u_\nu - u_\mu)\Bigr\|_{\mathbf{v}} \;\leq\; \sum_{\nu\neq\mu} w_\nu(\mathbf{v})\, d_g(\boldsymbol{\xi}_\nu, \boldsymbol{\xi}_\mu) \;\leq\; 2\, r_{\max} \sum_{\nu\neq\mu} w_\nu(\mathbf{v}).$$

Combining with (E.11) and Lemma E.6 yields (E.10). □

Remark E.8. The Lipschitz constant $L_{\mathrm{Exp}}(R(\mathbf{v}))$ appears only as a multiplicative prefactor in (E.10), and will therefore enter the capacity bound only through its logarithm. In the large-radius regime $\alpha r_{\max} \gg 1$, one has $\log L_{\mathrm{Exp}}(R(\mathbf{v})) = \Theta(\alpha r_{\max})$, which is of strictly lower order than the leading $d\, f_\kappa(r_{\min})$ term appearing below.

E.6. Chernoff Bound on the Margin Event

To control the margin event probabilistically, we first restrict to a high-probability set on which the radial norms are well-behaved.

Lemma E.9 (Noise control). *Let $\mathscr{E}_z := \{\|z\|_2 \leq \sqrt{2d}\}$. Then $\Pr[\mathscr{E}_z^c] \leq e^{-d/2}$, and on $\mathscr{E}_z$, $\|\mathrm{v}\|_2 \in [r^*_{\min}, r^*_{\max}]$ with*

$$r^*_{\min} := \max\{r_{\min} - \boldsymbol{\sigma}\sqrt{2d},\, 0\}, \qquad r^*_{\max} := r_{\max} + \boldsymbol{\sigma}\sqrt{2d}.$$

Moreover, under the noise condition

$$\boldsymbol{\sigma}\sqrt{2d} \;\leq\; r_{\min}/2, \tag{E.12}$$

we have $r^*_{\min} \geq r_{\min}/2 > 0$ and $R^* := r^*_{\max} + r_{\max} \leq 5r_{\max}/2$, so that $L_{\mathrm{Exp}}(R(\mathbf{v})) \leq L_{\mathrm{Exp}}(R^*)$ holds deterministically on $\mathscr{E}_z$.

Proof. The Gaussian-norm bound $\Pr[\|z\|_2 > \sqrt{2d}] \leq e^{-d/2}$ is a standard χ^2 tail bound (Wainwright, 2019). The remaining claims follow by the triangle inequality and from $g(r) = \sinh(\alpha r)/(\alpha r)$ being monotone increasing. □

We now combine Assumption E.5 with the Euclidean-to-hyperbolic bridge of Lemma D.2 to obtain a Chernoff bound on the margin event.

Proposition E.10 (Hyperbolic Chernoff)**.** *Suppose Assumption E.5 and the noise bound (E.12) hold. Define the inflated curvature parameters*

$$\gamma_\kappa^{\mathrm{H}} := f_\kappa(r^*_{\min})\,\gamma^{\mathrm{E}} - \widetilde{\mathsf{Pen}}_\kappa(r^*_{\min}, r^*_{\max})/d, \qquad K_\kappa^{\mathrm{H}} := f_\kappa(r^*_{\min})^2\, K^{\mathrm{E}}\,\boldsymbol{\sigma}^2. \tag{E.13}$$

Then for any $\Gamma < \gamma_\kappa^{\mathrm{H}} d$,

$$\Pr\big[\mathscr{M}_\mu^{\mathrm{H}}(\Gamma)^c \mid \mu,\, \mathscr{E}_z\big] \;\le\; (M-1)\,\exp\!\left(-\frac{(\gamma_\kappa^{\mathrm{H}} d - \Gamma)^2}{2K_\kappa^{\mathrm{H}} d}\right). \tag{E.14}$$

Proof. On $\mathscr{E}_z$, by Lemma D.2 applied with the inflated radii $[r^*_{\min}, r^*_{\max}]$,

$$\Delta_{\mu\nu}^{\mathrm{H}}(\mathbf{v}) \;\ge\; f_\kappa(r^*_{\min})\,\Delta_{\mu\nu}^{\mathrm{E}}(\mathtt{v}) \;-\; \widetilde{\mathsf{Pen}}_\kappa(r^*_{\min}, r^*_{\max}). \tag{E.15}$$

Define the (sub-Gaussian) surrogate

$$\widetilde{\Delta}_{\mu\nu}^{\mathrm{H}} \;:=\; f_\kappa(r^*_{\min})\,\Delta_{\mu\nu}^{\mathrm{E}}(\mathtt{v}) \;-\; \widetilde{\mathsf{Pen}}_\kappa(r^*_{\min}, r^*_{\max}),$$

so that $\Delta_{\mu\nu}^{\mathrm{H}} \ge \widetilde{\Delta}_{\mu\nu}^{\mathrm{H}}$ on $\mathscr{E}_z$. Because $\widetilde{\Delta}_{\mu\nu}^{\mathrm{H}}$ is a deterministic affine function of $\Delta_{\mu\nu}^{\mathrm{E}}(\mathtt{v})$, Assumption E.5 yields

$$\mathbb{E}\big[\widetilde{\Delta}_{\mu\nu}^{\mathrm{H}} \mid \mu\big] \;\ge\; f_\kappa(r^*_{\min})\,\gamma^{\mathrm{E}}\,d - \widetilde{\mathsf{Pen}}_\kappa(r^*_{\min}, r^*_{\max}) \;=\; \gamma_\kappa^{\mathrm{H}}\,d,$$

and $\widetilde{\Delta}_{\mu\nu}^{\mathrm{H}} - \mathbb{E}[\widetilde{\Delta}_{\mu\nu}^{\mathrm{H}} \mid \mu]$ is sub-Gaussian with proxy variance $K_\kappa^{\mathrm{H}}\,d$. The standard one-sided sub-Gaussian Chernoff bound gives, for any $\Gamma < \gamma_\kappa^{\mathrm{H}} d$,

$$\Pr\big[\widetilde{\Delta}_{\mu\nu}^{\mathrm{H}} < \Gamma \mid \mu\big] \;\le\; \exp\!\left(-\frac{(\gamma_\kappa^{\mathrm{H}} d - \Gamma)^2}{2K_\kappa^{\mathrm{H}} d}\right).$$

A union bound over $\nu \ne \mu$, combined with $\Delta_{\mu\nu}^{\mathrm{H}} \ge \widetilde{\Delta}_{\mu\nu}^{\mathrm{H}}$ on $\mathscr{E}_z$, yields (E.14). □

E.7. Capacity Bound

We now combine the above two steps to control the total recall error. We first present a more general result below (Theorem E.11), where Theorem 4.8 is a direct corollary.

Theorem E.11 (Capacity)**.** *Suppose Assumption E.5 and the noise bound (E.12) hold, that $\widetilde{\mathsf{Pen}}_\kappa(r^*_{\min}, r^*_{\max}) = o(d\, f_\kappa(r_{\min}))$, and that the SNR condition*

$$\boldsymbol{\sigma}^2\, f_\kappa(r^*_{\min}) \;=\; O(1) \quad\Longleftrightarrow\quad \boldsymbol{\sigma} \;=\; O\Big(\sqrt{|\kappa|}\, r_{\min}\, e^{-\alpha r_{\min}}\Big) z \tag{E.16}$$

holds. Then, as $d \to \infty$, the admissible number of stored patterns satisfies

$$\log M \;=\; \Theta\big(d\, f_\kappa(r_{\min})\big) \;-\; \widetilde{\mathsf{Pen}}_\kappa(r_{\min}, r_{\max}) \;=\; \Theta\!\left(\frac{d}{|\kappa|}\left(\frac{\sinh(\alpha r_{\min})}{r_{\min}}\right)^2\right) \;-\; \widetilde{\mathsf{Pen}}_\kappa. \tag{E.17}$$

Proof. By Proposition E.7, on $\mathscr{M}_\mu^{\mathrm{H}}(\Gamma) \cap \mathscr{E}_z$ the recall succeeds (i.e. $d_g(H(\mathbf{v}), \boldsymbol{\xi}_\mu) \le \varepsilon$) provided

$$\Gamma \;\ge\; \Gamma_\star(M) \;:=\; \log\!\left(\frac{2\, r_{\max}\, L_{\mathrm{Exp}}(R^*)\,(M-1)}{\varepsilon}\right) \;=\; \log M + O(\alpha r_{\max}). \tag{E.18}$$

A union bound over recall failure events gives

$$\Pr[\text{recall fail}] \;\le\; e^{-d/2} \;+\; (M-1)\,\exp\!\left(-\frac{(\gamma_\kappa^{\mathrm{H}} d - \Gamma_\star(M))^2}{2K_\kappa^{\mathrm{H}} d}\right). \tag{E.19}$$

Set $\log M = c\,\gamma_\kappa^{\mathrm{H}} d$ for some $c \in (0,1)$ to be chosen. Then $\Gamma_\star(M) = \log M + O(\alpha r_{\max})$, and the second term in (E.19) has exponent

$$\frac{(1-c)^2(\gamma_\kappa^{\mathrm{H}})^2 d}{2K_\kappa^{\mathrm{H}}}(1+o(1)) \;=\; \frac{(1-c)^2}{2}\,\frac{\gamma_\kappa^{\mathrm{H}}}{K_\kappa^{\mathrm{H}}}\,\gamma_\kappa^{\mathrm{H}} d\,(1+o(1)).$$

Vanishing total error therefore requires

$$c \;<\; \frac{(1-c)^2}{2}\,\frac{\gamma^{\mathrm{H}}_\kappa}{K^{\mathrm{H}}_\kappa}, \tag{E.20}$$

which admits a positive-constant solution c if and only if $\gamma^{\mathrm{H}}_\kappa/K^{\mathrm{H}}_\kappa = \Omega(1)$. By (E.13), this ratio is $\gamma^{\mathrm{H}}_\kappa/K^{\mathrm{H}}_\kappa \sim \gamma^{\mathrm{E}}/(f_\kappa(r^*_{\min})K^{\mathrm{E}}\boldsymbol{\sigma}^2)$, which is $\Omega(1)$ precisely under the SNR condition (E.16). Substituting $\gamma^{\mathrm{H}}_\kappa \sim f_\kappa(r_{\min})\gamma^{\mathrm{E}}$ (using $\widetilde{\mathsf{Pen}}_\kappa = o(d\,f_\kappa(r_{\min}))$) into $\log M = c\,\gamma^{\mathrm{H}}_\kappa d$ yields (E.17). □

Remark E.12. The Lipschitz constant $L_{\mathrm{Exp}}(R^*)$ contributes only an additive $O(\alpha r_{\max})$ term to $\Gamma_\star(M)$, which is dominated by the leading $d\,f_\kappa(r_{\min})$ term. Equivalently, the prefactor $e^{\alpha R^*}$ from L_{Exp} enters the capacity bound only after taking a logarithm: an exponentially-bad multiplicative factor in the recall error contracts to a linear additive cost in $\log M$. This is the key reason why the double-exponential scaling in $r_{\min}$ is preserved.

Corollary E.13. *Let $\kappa < 0$ and $\alpha = \sqrt{|\kappa|}$. Suppose Assumption E.5 and the noise bound (E.12) hold, that the stored patterns lie in a narrow shell with $\alpha r_{\min} \gg 1$ and*

$$\Delta_r \;=\; o\!\left(\frac{d}{\alpha\, r^2_{\min}}\right), \tag{E.21}$$

and that the SNR condition

$$\boldsymbol{\sigma} \;=\; O\!\left(\sqrt{|\kappa|}\, r_{\min}\, e^{-\alpha r_{\min}}\right) \tag{E.22}$$

holds. Then, as $d \to \infty$, the admissible number of stored patterns satisfies

$$\log M \;=\; \Theta\!\left(\frac{d}{|\kappa|}\,\frac{e^{2\alpha r_{\min}}}{r^2_{\min}}\right), \qquad M \;=\; \exp\!\left(\Theta\!\left(\frac{d}{|\kappa|}\,\frac{e^{2\alpha r_{\min}}}{r^2_{\min}}\right)\right). \tag{E.23}$$

Proof. The width condition (E.21) ensures $\widetilde{\mathsf{Pen}}_\kappa(r_{\min}, r_{\max}) = o(d\,f_\kappa(r_{\min}))$ by Corollary E.17, so the hypotheses of Theorem E.11 are satisfied. In the large-radius regime $\alpha r_{\min} \gg 1$,

$$f_\kappa(r_{\min}) \;=\; \frac{1}{2|\kappa|}\left(\frac{\sinh(\alpha r_{\min})}{r_{\min}}\right)^2 \;=\; \Theta\!\left(\frac{1}{|\kappa|}\,\frac{e^{2\alpha r_{\min}}}{r^2_{\min}}\right).$$

Substituting into (E.17) and absorbing the penalty into the lower-order term yields (E.23). □

Remark E.14. We refer to (E.23) as *double-exponential capacity*: M is exponential in d, and the rate of that exponential is itself exponential in the radius $r_{\min}$. This radial amplification is a strictly hyperbolic phenomenon, with no analogue in Euclidean associative memory models (Ramsauer et al., 2020; Krotov & Hopfield, 2021), where capacity scales at most exponentially in d with a curvature-independent rate.

Remark E.15. Corollary E.13 clarifies the role of each hypothesis. The width condition (E.21) controls the bridge penalty so that the hyperbolic margin inherits the sub-Gaussian Chernoff exponent of the Euclidean gap. The SNR condition (E.22) ensures that the variance proxy $K^{\mathrm{H}}_\kappa = f_\kappa(r^*_{\min})^2 K^{\mathrm{E}}\boldsymbol{\sigma}^2$ does not absorb the entire amplification. Together, they pin the capacity rate to $f_\kappa(r_{\min})$, which carries the double-exponential growth.

E.8. Capacity Regimes

The capacity rate (E.17) admits several natural regimes, summarized in Table 3.

We now isolate the two regimes of primary interest.

Corollary E.16 (Thin shell)**.** *Suppose $r_{\min} = r_{\max} = r$, so that $\Delta_r = 0$ and $\widetilde{\mathsf{Pen}}_\kappa = 0$. Then under the conditions of Theorem E.11,*

$$\log M \;=\; \Theta\!\left(\frac{d}{|\kappa|}\left(\frac{\sinh(\alpha r)}{r}\right)^2\right). \tag{E.24}$$

In the small-radius regime $\alpha r \ll 1$, this reduces to $\log M = \Theta(d)$. In the large-radius regime $\alpha r \gg 1$,

$$\log M \;=\; \Theta\!\left(\frac{d}{|\kappa|}\,\frac{e^{2\alpha r}}{r^2}\right),$$

exhibiting double-exponential radial amplification: $M = \exp\!\big(\Theta(d\,e^{2\alpha r}/(|\kappa| r^2))\big)$.

Table 3. Capacity scaling regimes under the hyperbolic Chernoff analysis. Here $\alpha = \sqrt{|\kappa|}$, $r = r_{\min} = r_{\max}$ in the thin-shell regime, and $\Delta_r = r_{\max} - r_{\min}$ otherwise.

Regime	Δ_r	$\widetilde{\mathsf{Pen}}_\kappa$	$\log M$
Thin shell	0	0	$\Theta\big(d\, f_\kappa(r)\big) = \Theta\big(\frac{d}{\lvert\kappa\rvert}\,\frac{e^{2\alpha r}}{r^2}\big)$
Narrow shell	$O(1/d)$	$O(1)$	$\Theta\big(d\, f_\kappa(r_{\min})\big)$
Sub-critical shell	$o\big(\frac{d}{\alpha r_{\min}^2}\big)$	$o(d\, f_\kappa)$	$\Theta\big(d\, f_\kappa(r_{\min})\big)$
Super-critical shell	$\Omega\big(\frac{d}{\alpha r_{\min}^2}\big)$	$\Omega(d\, f_\kappa)$	**Collapse**

Corollary E.17 (Width–amplification tradeoff)**.** *Exponential-in-d capacity persists if and only if $\widetilde{\mathsf{Pen}}_\kappa(r_{\min}, r_{\max}) = o(d\, f_\kappa(r_{\min}))$. In the large-radius regime $\alpha r_{\min} \gg 1$, this condition is implied by*

$$\Delta_r \;=\; o\biggl(\frac{d}{\alpha\, r_{\min}^2}\biggr), \tag{E.25}$$

showing that admissible radial spread is at most of order $d/(\alpha r_{\min}^2)$ before the penalty term dominates and capacity collapses.

Remark E.18. The SNR condition (E.16) admits a natural interpretation as a noise-to-curvature balance: in order for the hyperbolic amplification factor f_κ to transfer fully into capacity, the noise $\boldsymbol{\sigma}$ must shrink at most inverse-exponentially with $\alpha r_{\min}$. Combined with the milder condition (E.12), this is the binding constraint as $\alpha r_{\min} \to \infty$.

E.9. Derivations

E.9.1. Derivation of Posterior

By Bayes' rule, the posterior over memory index μ is

$$p(\mu \mid \boldsymbol{n}) = \frac{p(\boldsymbol{n} \mid \mu)\, p(\mu)}{p(\boldsymbol{n})}.$$

Taking logarithms,

$$\log p(\mu \mid \boldsymbol{n}) = \log p(\boldsymbol{n} \mid \mu) + \log p(\mu) - \log p(\boldsymbol{n}).$$

Assuming conditional independence of the spike counts across neurons given μ, the Poisson log-likelihood gives

$$\log p(\boldsymbol{n} \mid \mu) = \sum_{i=1}^{N} \bigl(n_i \log \lambda_i^\mu - \lambda_i^\mu \Delta t\bigr) + C_1,$$

where C_1 collects terms (e.g. factorials $\log(n_i!)$) that do not depend on μ when the rates $\{\lambda_i^\mu\}$ are fixed. Hence

$$\log p(\mu \mid \boldsymbol{n}) = \sum_{i=1}^{N} \bigl(n_i \log \lambda_i^\mu - \lambda_i^\mu \Delta t\bigr) + \log p(\mu) - \log p(\boldsymbol{n}) + C_1.$$

Equivalently, absorbing the μ-independent factor $e^{C_1}/p(\boldsymbol{n})$ into normalization,

$$p(\mu \mid \boldsymbol{n}) \propto \exp\Bigl(\sum_{i=1}^{N} \bigl(n_i \log \lambda_i^\mu - \lambda_i^\mu \Delta t\bigr) + \log p(\mu)\Bigr).$$

E.9.2. Derivation of (2.6)

Proof. Write the log-posterior scores

$$h_\mu(\boldsymbol{n}) := \log p(\boldsymbol{n} \mid \mu) + \log p(\mu),$$

so that $\log p(\mu \mid \boldsymbol{n}) = h_\mu(\boldsymbol{n}) - \log p(\boldsymbol{n})$. Then

$$\begin{aligned}
\exp\bigl(\log p(\mu \mid \boldsymbol{n})\bigr) &= \exp\bigl(h_\mu(\boldsymbol{n}) - \log p(\boldsymbol{n})\bigr) \\
&= \exp\bigl(h_\mu(\boldsymbol{n})\bigr)\ \exp\bigl(-\log p(\boldsymbol{n})\bigr) \\
&= \frac{\exp\bigl(h_\mu(\boldsymbol{n})\bigr)}{\exp\bigl(\log p(\boldsymbol{n})\bigr)} \\
&= \frac{\exp\bigl(h_\mu(\boldsymbol{n})\bigr)}{p(\boldsymbol{n})}.
\end{aligned}$$

Summing over all μ and using $\sum_\nu p(\nu \mid \boldsymbol{n}) = 1$,

$$1 = \sum_\nu \frac{\exp\bigl(h_\nu(\boldsymbol{n})\bigr)}{p(\boldsymbol{n})},$$

so $p(\boldsymbol{n}) = \sum_\nu \exp(h_\nu(\boldsymbol{n}))$. Finally, let $\boldsymbol{h} \coloneqq \bigl(h_1(\boldsymbol{n}), \ldots, h_M(\boldsymbol{n})\bigr)$. Then

$$p(\mu \mid \boldsymbol{n}) = \mathrm{softmax}_\mu(\boldsymbol{h}) = \frac{\exp\bigl(h_\mu(\boldsymbol{n})\bigr)}{\sum_{\nu=1}^{M} \exp\bigl(h_\nu(\boldsymbol{n})\bigr)}.$$

□

F. Simulation Details

F.1. Pattern Completion

Baseline Implementation. We implement two associative memory models, the modern Hopfield networks (Ramsauer et al., 2020) and dense associative memory (Krotov & Hopfield, 2021). For MHN, its update rule is

$$v^{(\text{new})} \leftarrow \sum_{\mu=1}^{M} \text{softmax}_\mu \left(\beta v^\top \xi_1, \cdots, \beta v^\top \xi_M\right).$$

For DAM, its update rule is

$$v^{(\text{new})} \leftarrow \sum_{\mu=1}^{M} w_\mu \cdot \xi_\mu, \quad w_\mu = (\beta v^\top \xi_\mu)^n.$$

To compute the posterior for DAM, we have

$$p(\mu \mid v) = \frac{w_\mu}{\sum_\nu w_\nu}.$$

Sampling. For synthetic memory patterns, we uniformly sample them inside a ball with radius $r_{\max}$. To do so, we adopt a common approach by sampling the point direction and radius separately.

Preprocessing. For data preprocessing, we only use `torch.ToTensor` to normalize pixels to $[0, 1]$. Next, for real-world images, we apply PCA to reduce their dimensionality to some d, we then rescale the memory patterns with $r_{\max}$. For KFM, we project them to the hyperboloid model by first concatenating an extra dimension to the first entry, with value 1 (totangent), then apply the exponential map.

Hyperparameters. The hyperparameters are listed in Table 4. Specifically, the major difference between the synthetic data and real-world data is we use $\beta = 1.0$ for synthetic data, and $\beta = 10.0$ for real-world data. For all results, we report the mean and standard deviation across 10 runs.

Table 4. Hyperparameter used in the pattern completion task.

**parameter**		**Value**	
**Dataset**	MNIST	CIFAR10	Synthetic
max. update steps	64	64	64
M	$[10, 1000]$	$[10, 1000]$	$[10, 1000]$
d	$\{10, 20, 100\}$	$\{10, 20, 100\}$	$\{10, 20, 100\}$
β	1	10	10
ϵ	0.01	0.01	0.01
$r_{\max}$	3	3	3
DAM order	10	10	10

F.2. Multiple Instance Learning

Here we briefly review the concept of multiple instance learning (MIL). MIL is a variant of supervised learning in which the training set consists of labeled bags containing multiple instances. The objective is to predict bag-level labels from the instances within each bag, making MIL particularly suitable for settings where instance-level annotation is difficult or impractical but bag-level labels are available. Applications include medical imaging, where bags correspond to images and instances to image patches, and document classification, where bags correspond to documents and instances to words or sentences. The statistics of the benchmark dataset we used in the paper is in Table 5.

Table 5. Statistics of MIL benchmark datasets

Name	Instances	Features	Bags	+bags	−bags
Elephant	1391	230	200	100	100
Fox	1302	230	200	100	100
Tiger	1220	230	200	100	100

F.3. Machine Learning Layers

Layer Definition The hyperbolic attention layer maps Euclidean inputs to a Hadamard manifold $\mathcal{M} = \mathbb{H}^d_\kappa$ for associative recall and returns the Euclidean mappings. Let $R \in \mathbb{R}^{S\times d}$ and $Y \in \mathbb{R}^{M\times d}$ denote the state and memory matrices, with learned weights $W_Q, W_K, W_V \in \mathbb{R}^{d\times d}$. Inputs are projected into $\mathcal{M}$ via the exponential map $\text{Exp}_{\boldsymbol{o}}$ at the origin $\boldsymbol{o} = [\sqrt{k}, 0]$:

$$\begin{aligned} \boldsymbol{Q} &= \text{Exp}_o(RW_Q), \\ \boldsymbol{K} &= \text{Exp}_o(YW_K), \\ \boldsymbol{V} &= \text{Exp}_o(YW_KW_V). \end{aligned} \tag{F.1}$$

The attention weights are determined by the Minkowski inner product $\langle \boldsymbol{q}_i, \boldsymbol{k}_j\rangle_L$ such that $\alpha_{ij} = \text{softmax}_j\left(-\beta\langle \boldsymbol{q}_i, \boldsymbol{k}_j\rangle_L\right)$.

Then, the manifold output $\boldsymbol{Z} \in \mathcal{M}^{S\times d}$ is computed via Karcher flow, approximating the weighted Fréchet Mean of $\boldsymbol{V}$ through the update:

$$\boldsymbol{z}_i = \text{Exp}_{\boldsymbol{q}_i}\left(\sum_{j=1}^{M} \alpha_{ij}\text{Exp}^{-1}_{\boldsymbol{q}_i}(\boldsymbol{v}_j)\right). \tag{F.2}$$

where $\boldsymbol{Z} = [\boldsymbol{z}_1, \boldsymbol{z}_2, \cdots, \boldsymbol{z}_S]$.

Types of layers The general hyperbolic attention mechanism described above can be specialized into three distinct layer types by specifying different sources of the state R and memory Y. These layers are the hyperbolic analogs of the `Hopfield`, `HopfieldPooling`, and `HopfieldLayer` modules described in (Ramsauer et al., 2020).

Karcher Flow Attention: This is the implentation of (F.2). Both R and Y are dynamic inputs from preceding layers or another input source. This configuration performs hyperbolic self-attention or cross-attention between two sets of vectors.

Karcher Flow Pooling: In this layer, patterns propagate via the memory patterns Y. The stored memories Y are summarized through queries by the static patterns $\Xi \in \mathbb{R}^{S\times d}$. We define $\alpha_{ij} = \text{softmax}_j\left(-\beta\langle \boldsymbol{\xi}_i, \boldsymbol{k}_j\rangle_L\right)$ and the update rule for this layer is given by:

$$\boldsymbol{z}_i = \text{Exp}_{\boldsymbol{\xi}_i}\left(\sum_{j=1}^{M} \alpha_{ij}\text{Exp}^{-1}_{\boldsymbol{\xi}_i}(\boldsymbol{v}_j)\right). \tag{F.3}$$

where $\boldsymbol{\Xi} = \text{Exp}_{\boldsymbol{o}}(\Xi)$.

Karcher Flow Layer: In this layer, patterns propagate via the state patterns R. Memories are fixed and represented by the weight matrix $W_K \in \mathbb{R}^{M\times d}$. The input R acts as the query set. We define $\alpha_{ij} = \text{softmax}_j\left(-\beta\langle \boldsymbol{r}_i, (\boldsymbol{W}_K)_j\rangle_L\right)$ and the update rule for this layer is given by:

$$\boldsymbol{z}_i = \text{Exp}_{\boldsymbol{r}_i}\left(\sum_{j=1}^{M} \alpha_{ij}\text{Exp}^{-1}_{\boldsymbol{r}_i}((\boldsymbol{W}_V)_j)\right). \tag{F.4}$$

where $\boldsymbol{R} = \text{Exp}_{\boldsymbol{o}}(R)$, $\boldsymbol{W}_K = \text{Exp}_{\boldsymbol{o}}(W_K)$, and $\boldsymbol{W}_V = \text{Exp}_{\boldsymbol{o}}(W_V)$.

Table 6. Hyperparameters used in attention tasks.

**Parameter**	**MNIST**	**MIL**
Optimizer	AdamW	AdamW
Learning Iteration N	14	100
Batch Size	64	16
Update Rule Iteration	1	1
Learning Rate	0.001	0.001
Learning Rate Decay (γ)	0.96	0.96
LR Scheduler	Step Decay	Step Decay
Hidden Dimension d	$\{4, 8, 32\}$	128
Scaling Factor (β)	$\frac{1}{\sqrt{d}}$	$\frac{1}{\sqrt{128}}$
Bag Dropout	—	0.5

G. Additional Simulations

G.1. Pattern Completion

Here we further investigate the case of how increasing $r_{\max}$ would help memory storage (pattern separation). Specifically, we now evaluate this property on real world datasets. We follow the style in Figure 2(b) and plot the model performance under different values of $r_{\max}$ in the same subplot. We observe that not only did KFM performs the best when $d = 10$. We can also observe that, on the tail of the performance curve (when M is large), only KFM is able to gain notable performance boost. Meanwhile, other baseline models still shows a rapid recall rate decay when M is close to 1000.

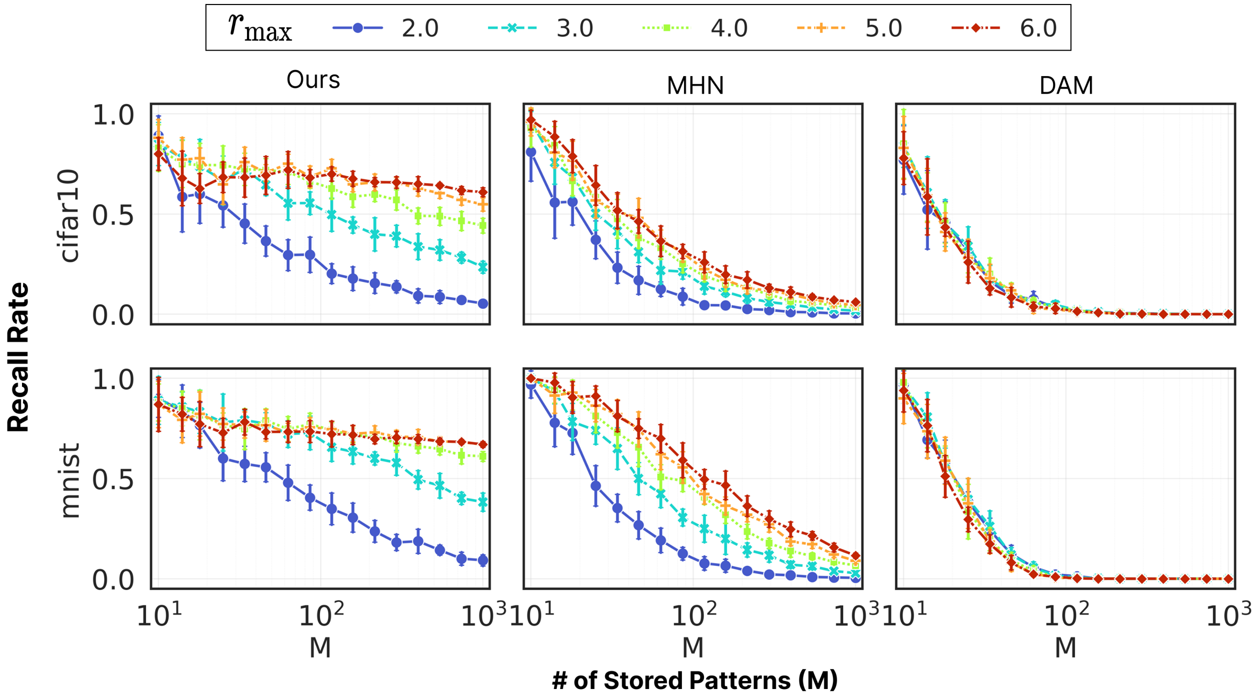

Figure 4. **Recall success rate under different values of $r_{\max}$. Columns left-to-right: KFM, MHN, DAM.** To further validate our double-exponential capacity result in $r_{\max}$, we perform pattern completion on different datasets, rescaling the images to different $r_{\max}$. We observe that KFM achieves the best recall rate when M is large. In particular, at $M = 1000$, KFM is the only model with substantial capacity improvement. Here we set the PCA dimension to 10 for all models and datasets.

G.2. Hyperbolicity Simulation

Here we empirically compute the hyperbolicity constant δ when the neural representation is generated from different tuning curve families. Specifically, we define 5 tuning curves under different place field size distributions, and see how their empirical δ scales with respect to the size of the environment. We define their probability density functions and mean values as follows. Specifically, we choose the parameters carefully so that all distributions has the same mean values. The results are in Figure 5. We observe that both the exponential distribution and the log-normal distributions remain statistically hyperbolic when L increases, which are the two distributions reported in (Zhang et al., 2023). Both constant and uniform distributions are not able to remain statistically hyperbolic as their delta grows significantly with L. Interestingly, the half-normal distribution is able to also maintain low δ, but is slightly less hyperbolic than the exponential and log-normal distributions.

Exponential.

$$X \sim \text{Exp}(\lambda = 1), \quad f_X(x) = \begin{cases} e^{-x}, & x \geq 0 \\ 0, & x < 0 \end{cases}, \quad \mathbb{E}[X] = 1.$$

Log-normal.

$$X \sim \mathrm{LogNormal}(\mu = -0.5, \sigma = 1), \quad f_X(x) = \frac{1}{x\sqrt{2\pi}} \exp\left(-\frac{(\ln x + 0.5)^2}{2}\right), \qquad x > 0, \quad \mathbb{E}[X] = e^{\mu + \sigma^2/2} = 1.$$

Uniform.

$$X \sim \mathrm{Uniform}(0.2, 1.8), \quad f_X(x) = \begin{cases} \frac{1}{1.6}, & 0.2 \le x \le 1.8 \\ 0, & \text{otherwise} \end{cases}, \quad \mathbb{E}[X] = \frac{0.2 + 1.8}{2} = 1.$$

Constant.

$$X = 1, \quad f_X(x) = Dirac(x - 1),$$

where $Dirac$ denotes the Dirac delta distribution.

Half-normal.

$$X \sim \mathrm{HalfNormal}\left(\sigma = \sqrt{\frac{\pi}{2}}\right), \quad f_X(x) = \sqrt{\frac{2}{\pi\sigma^2}} \exp\left(-\frac{x^2}{2\sigma^2}\right), \qquad x \ge 0, \quad \sigma = \sqrt{\frac{\pi}{2}}, \quad \mathbb{E}[X] = \sigma\sqrt{\frac{2}{\pi}} = 1.$$

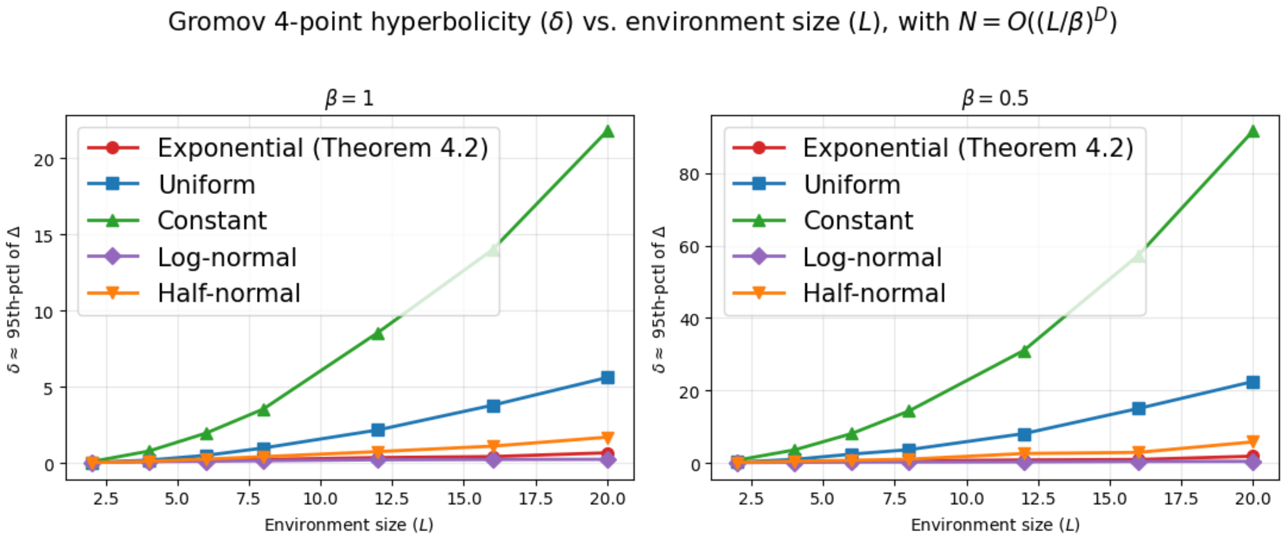

Figure 5. **Empirical hyperbolicity under different place field size distributions.** We observe that both the exponential distribution and the log-normal distributions remain statistically hyperbolic when L increases, which are the two distributions reported in (Zhang et al., 2023). Both constant and uniform distributions are not able to remain statistically hyperbolic as their delta grows significantly with L. Interestingly, the half-normal distribution is able to also maintain low δ, but is slightly less hyperbolic than the exponential and log-normal distributions.
